# The predictive validity for mortality of the driving pressure and the mechanical power of ventilation

**DOI:** 10.1186/s40635-020-00346-8

**Published:** 2020-12-18

**Authors:** David M. P. van Meenen, Ary Serpa Neto, Frederique Paulus, Coen Merkies, Laura R. Schouten, Lieuwe D. Bos, Janneke Horn, Nicole P. Juffermans, Olaf L. Cremer, Tom van der Poll, Marcus J. Schultz, for the MARS Consortium, Friso M. de Beer, Lieuwe D. Bos, Gerie J. Glas, Janneke Horn, Arie J. Hoogendijk, Roosmarijn T. van Hooijdonk, Mischa A. Huson, Tom van der Poll, Brendon Scicluna, Laura R. Schouten, Marcus J. Schultz, Marleen Straat, Lonneke A. van Vught, Luuk Wieske, Maryse A. Wiewel, Esther Witteveen, Marc J. Bonten, Olaf L. Cremer, Jos F. Frencken, Kirsten van de Groep, Peter M. Klein Klouwenberg, Maria E. Koster-Brouwer, David S. Ong, Meri R. Varkila, Diana M. Verboom

**Affiliations:** 1 Department of Intensive Care, University of Amsterdam, Amsterdam University Medical Centers, Location “Academic Medical Center”, Meibergdreeg 9, 1105 AZ Amsterdam, The Netherlands; 2 Department of Critical Care Medicine, Hospital Israelita Albert Einstein, Av. Albert Einstein, 627 – Morumbi, São Paulo, Brazil; 3 Laboratory of Experimental Intensive Care and Anesthesiology (L·E·I·C·A), University of Amsterdam, Amsterdam University Medical Centers, Location “Academic Medical Center”, Meibergdreeg 9, 1105 AZ Amsterdam, The Netherlands; 4 Department of Intensive Care Medicine, University Medical Center Utrecht, Heidelberglaan 100, 3584 CX Utrecht, The Netherlands; 5 Center for Experimental and Molecular Medicine (CEMM), University of Amsterdam, Amsterdam University Medical Centers, Location “Academic Medical Center”, Meibergdreeg 9, 1105 AZ Amsterdam, The Netherlands; 6 Center for Infection and Immunity Amsterdam, University of Amsterdam, Amsterdam University Medical Centers, Location “Academic Medical Center”, Meibergdreeg 9, 1105 AZ Amsterdam, The Netherlands; 7 Division of Infectious Diseases, University of Amsterdam, Amsterdam University Medical Centers, Location “Academic Medical Center”, Meibergdreeg 9, 1105 AZ Amsterdam, The Netherlands; 8 Mahidol–Oxford Tropical Medicine Research Unit (MORU), Mahidol University, 999 Phutthamonthon Sai 4 Rd, Bangkok, Thailand; 9 Nuffield Department of Medicine, University of Oxford, Oxford, OX1 2JD UK

**Keywords:** Intensive care unit, Invasive ventilation, Mortality, Prognostication, Predictive validity, Respiratory system driving pressure, Driving pressure, ΔP, Mechanical power of ventilation, Mechanical power

## Abstract

**Background:**

Outcome prediction in critically ill patients under invasive ventilation remains extremely challenging. The driving pressure (ΔP) and the mechanical power of ventilation (MP) are associated with patient-centered outcomes like mortality and duration of ventilation. The objective of this study was to assess the predictive validity for mortality of the ΔP and the MP at 24 h after start of invasive ventilation.

**Methods:**

This is a post hoc analysis of an observational study in intensive care unit patients, restricted to critically ill patients receiving invasive ventilation for at least 24 h. The two exposures of interest were the modified ΔP and the MP at 24 h after start of invasive ventilation. The primary outcome was 90-day mortality; secondary outcomes were ICU and hospital mortality. The predictive validity was measured as incremental 90-day mortality beyond that predicted by the Acute Physiology, Age and Chronic Health Evaluation (APACHE) IV score and the Simplified Acute Physiology Score (SAPS) II.

**Results:**

The analysis included 839 patients with a 90-day mortality of 42%. The median modified ΔP at 24 h was 15 [interquartile range 12 to 19] cm H_2_O; the median MP at 24 h was 206 [interquartile range 145 to 298] 10^−3^ J/min/kg predicted body weight (PBW). Both parameters were associated with 90-day mortality (odds ratio (OR) for 1 cm H_2_O increase in the modified ΔP, 1.05 [95% confidence interval (CI) 1.03 to 1.08]; *P* < 0.001; OR for 100 10^−3^ J/min/kg PBW increase in the MP, 1.20 [95% CI 1.09 to 1.33]; *P* < 0.001). Area under the ROC for 90-day mortality of the modified ΔP and the MP were 0.70 [95% CI 0.66 to 0.74] and 0.69 [95% CI 0.65 to 0.73], which was neither different from that of the APACHE IV score nor that of the SAPS II.

**Conclusions:**

In adult patients under invasive ventilation, the modified ΔP and the MP at 24 h are associated with 90 day mortality. Neither the modified ΔP nor the MP at 24 h has predictive validity beyond the APACHE IV score and the SAPS II.

## Introduction

Outcome prediction in intensive care unit (ICU) patients under invasive ventilation for acute respiratory failure is challenging [1, 2]. Disease severity scores, like the Acute Physiology, Age and Chronic Health Evaluation (APACHE) IV score, and the Simplified Acute Physiology Score (SAPS) II, are effective in estimating the risk of death in the general ICU population [3, 4]. For ICU patients with acute respiratory distress syndrome (ARDS), the Berlin Definition for ARDS has been proposed for risk of death classification, [5] albeit with limited success [6, 7].

The driving pressure (ΔP) represents the ratio between tidal volume (*V*_*T*_) and respiratory system compliance (*C*) [8, 9], and can be calculated as the difference between plateau pressure (*P*_plat_) and positive end-expiratory pressure (PEEP). The ΔP has been shown to be the ventilator parameter associated most strongly with mortality, and is even suggested as a key parameter for optimization when applying invasive ventilation [10–13]. The mechanical power of ventilation (MP) represents the amount of energy per unit of time transferred from the ventilator to the respiratory system and lung tissue [14], and can be calculated as the product of *V*_*T*_, respiratory rate (RR), and the difference between the peak pressure (*P*_peak_) and 0.5 × ΔP [15]. An independent association between the MP and mortality has been demonstrated in invasively ventilated ICU patients [16, 17].

The associations between the ΔP and the MP, and mortality make them both attractive for use in risk classification for death. It is unknown, however, whether the ΔP and the MP hold prognostic value, and in particular, whether they add to the frequently used and robust APACHE IV score and SAPS II. Therefore, we determined the predictive validity of the ΔP and the MP using data stored in the database of the “Molecular diAgnosis and Risk Stratification of sepsis” (MARS) study, an observational study that captured granular data of a cohort of general ICU patients in two Dutch hospitals [18, 19]. It was hypothesized that the ΔP and the MP, after initial ventilatory stabilization, are associated with 90-day mortality, and have predictive validity beyond the APACHE IV score and the SAPS II.

## Methods

### Design and ethical approval

This was a post hoc analysis of the MARS study that ran from January 2011 to January 2014. The database of the MARS study contains prospectively collected detailed demographic, clinical and outcome data, and detailed ventilator settings, variables, and parameters from a large cohort of ICU patients, not restricted to patients with sepsis but instead patients who were admitted beyond the next calendar day [18, 19]. The Institutional Review Board approved the protocol of MARS (protocol no. 10–056C) and the use of an opt-out consent procedure, in which participants and their legal representatives were notified of the study in writing. The MARS study was registered at www.clinicaltrials.gov (study identifier NCT01905033).

### Inclusion and exclusion criteria

Patients were eligible for inclusion in the MARS study if they had an expected length of stay in the ICU of > 24 h. The MARS study itself had no exclusion criteria. For the purpose of the current post hoc analysis, readmitted patients were excluded, as well as patients who never had received invasive ventilation, or had received invasive ventilation for < 24 h. To have reliable calculation of the modified ΔP and the MP, patients under pressure support ventilation, and patients in whom there was evidence of spontaneous breathing at 24 h after start of invasive ventilation were excluded, as were patients of whom we had incomplete data necessary for calculating the modified ΔP or the MP at that time point.

### Collection of data and diagnosing ARDS

In the MARS study, a dedicated team of trained researchers collected baseline characteristics and outcomes and diagnosed ARDS and its severity using the at that time used American–European Consensus Conference definition for ARDS. All patients could be reclassified using the Berlin Definition [20]; patients originally classified as having ARDS with the older definition could be re-classified as having mild, moderate, or severe ARDS with the latest definition.

### Calculation of the ΔP and the MP

Ventilation variables, including ventilatory mode, *V*_*T*_, set and measured RR (RR_set_ and RR_measured_), maximum airway pressure (*P*_max_) at zero flow, PEEP, fraction of inspired oxygen (FiO_2_), and blood gas analysis results were collected at the start of ventilation, after 24 h and thereafter daily till invasive ventilation was discontinued; measurements were collected for a single breath, and only if the patients was stable, and sufficiently long after certain procedures (like nursing activities, changes in body position, recruitment maneuvers if used). Spontaneous breathing was recognized by comparing RR_set_ and RR_measured_, i.e., a higher RR_measured_ than RR_set_ was seen as evidence of spontaneous breathing.

For calculating the modified ΔP and the MP, ventilation variables collected at 24 h after start of invasive ventilation were used, to guarantee that all patients were sufficiently stabilized, and also because previous studies showed that using ventilation data at that time point had better predictive capacities than those collected shortly after initiation of invasive ventilation [20–23].

The modified ΔP was calculated by subtracting PEEP from *P*_max_ [22, 24].

1 $$ \Delta \mathrm{P}={\mathrm{P}}_{\mathrm{max}}-\mathrm{PEEP} $$

Absolute MP was calculated using an adjusted power equation [15, 25].

2 $$ \mathrm{Absolute}\ \mathrm{MP}=0.098\ast {\mathrm{V}}_{\mathrm{T}}\ast \mathrm{RR}\ast \left(\mathrm{Pmax}-0.5\ast \Delta \mathrm{P}\right) $$

*P*_peak_ is suggested to be used in the originally reported “power equation” [25]. As proposed before [22, 24], for the present analysis *P*_max_ instead of *P*_peak_ was used, as in the participating ICUs pressure-controlled ventilation was exclusively used for assist ventilation.

Predicted body weight (PBW) was calculated by using the equation as used in previous studies of ventilation [26].

3a $$ \mathrm{PBW}=50+0\cdotp 91\ \left(\mathrm{height}\ \left[\mathrm{cm}\right]-152\cdotp 4\right)\ \mathrm{in}\ \mathrm{males} $$

3b $$ \mathrm{PBW}=45\cdotp 5+0\cdotp 91\ \left(\mathrm{height}\ \left[\mathrm{cm}\right]-152\cdotp 4\right)\ \mathrm{in}\ \mathrm{females} $$

The MP was normalized to PBW by dividing the absolute MP by the PBW [16].

4 $$ \mathrm{MP}=\mathrm{absolute}\ \mathrm{MP}/\mathrm{PBW} $$

### Outcomes

The following clinical outcomes were collected: 90-day mortality, ICU and hospital mortality, and duration of ventilation expressed as the number of days ventilator-free and alive at day 28 (VFD–28).

### Study endpoint

The primary study endpoint was the added predictive value of the two ventilation parameters of interest to baseline 90-day mortality prediction based on APACHE IV scores. Secondary endpoints were the odds ratios (ORs) of the two ventilation parameters of interest for 90-day, ICU and hospital mortality, and the effect estimates (EEs) for VFD–28.

### Statistical analysis

Continuous variables were expressed as medians (25th–75th interquartile range [IQR]), and categorical variables as proportions. Continuous variables were analyzed using Mann-Whitney *U* test for non-normally distributed data or a Welch two-sample *t* test for normally distributed data, proportions were compared using Fisher exact test.

First, patients were scored based on the modified ΔP and the MP calculated at 24 h after the start of invasive ventilation in the ICU. Based on the median of the modified ΔP and the MP, patients were stratified into groups of patients with a low or a high modified ΔP, and a low or a high MP, respectively. Survival analyses were performed for the four groups using a log-rank test. Then, the ORs for an increase 1 cm H_2_O in the modified ΔP, and for an increase in 100 10^−3^ J/min/kg PBW in the MP for 90-day mortality, ICU mortality, and hospital mortality were calculated. The EEs for VFD–28 were also calculated.

For determining the predictive validity of the two ventilatory parameters of interest, the first baseline risk for death was calculated using a generalized linear model in which 90-day mortality was used as outcome and the APACHE IV score or SAPS II as predictor. Thereafter, two separate models were created in which the modified ΔP and the MP were added. This data was then used to calculate the area under the receiver operator characteristic curve (AUROC) of in total three models (i.e., the baseline risk, the baseline risk + the modified ΔP, and the baseline risk + the MP).

One sensitivity analysis was performed, in which the interaction between the predictive validity of the ΔP or the MP for 90-day mortality and presence of ARDS was determined. As a post hoc analysis, we calculated the AUROC of an additional model containing both modified ΔP and MP.

Statistical analyses were performed using R and the R–Studio interface (R version 3.3.3., www.r-project.org, Vienna, Austria, retrieved January 2017). A *P* value < 0.05 was considered as statistically significant.

## Results

### Patients

**Fig. 1:**
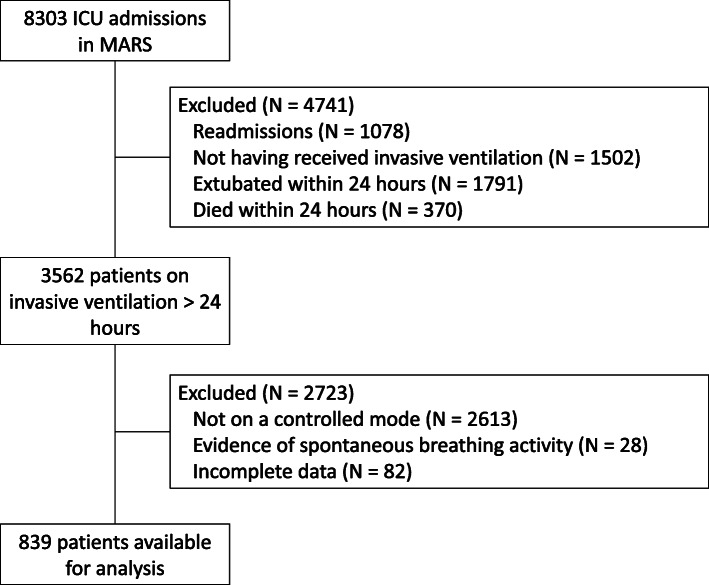
Flow chart of inclusion of patients. Abbreviations: ICU, intensive care unit; MARS, “Molecular diAgnosis and Risk Stratification”

Consort diagram is given in Fig. 1. Of the 8303 enrollments in the MARS study, 3562 (43%) patients were under invasive ventilation for > 24 h. After excluding ineligible patients, a total of 839 patients were selected for the current analysis. Baseline demographics, major outcome data and ventilation settings, variables, and parameters are presented in Table 1. Baseline characteristics of patients with a low versus a high modified ΔP, and a low versus a high MP are presented in Table 1. All-cause 90-day mortality was 42%. Survivors and non-survivors differed in age, risk of death based on the APACHE IV score, presence of chronic kidney failure, blood pressure, body temperature, and sequential organ failure assessment (SOFA) scores. Non-survivors had less VFD–28, a shorter hospital length of stay (LOS), and higher ICU and hospital mortality rates. FiO_2_ and PEEP were similar between survivors and non-survivors; *P*_max_ was lower, and pHa and arterial HCO_3_^−^ levels were higher in survivors compared to non-survivors.

### The ΔP and the MP in survivors and non-survivors

**Fig. 2:**
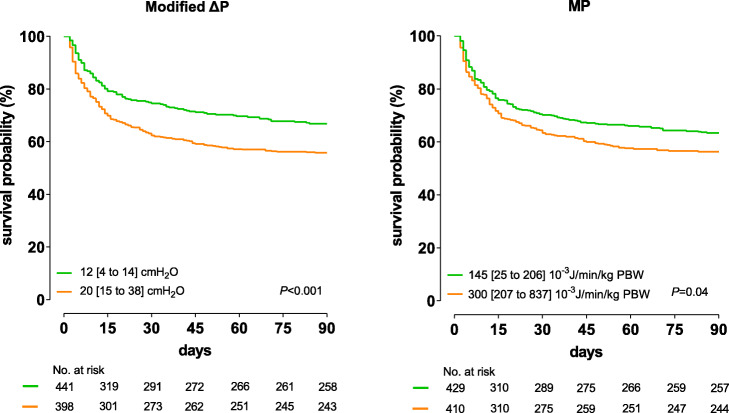
Kaplan-Meier curves for 90-day mortality for low and high modified ΔP and low and high MP groups. Curves were compared using a log-rank test. Abbreviations: ΔP, respiratory system driving pressure; MP, mechanical power corrected for predicted body weight; PBW, predicted body weight

The median modified ΔP was 15 [12 to 19] cm H_2_O; the median MP was 206 [145 to 298] 10^−3^ J/min/kg PBW. Based on these, 441 patients were stratified to a low modified ΔP group, and 398 were stratified to the high modified ΔP group; the low MP group consisted of 429 patients, and the high MP group of 410 patients. The modified ΔP and the MP were significantly lower in survivors than in non-survivors (Table 1). Kaplan-Meier curves for the patients with a low or high modified ΔP, and a low or high MP are shown in Fig. 2.

**Table 1:** Patient characteristics, outcomes, and ventilation characteristics

Characteristics	All	Survivors	Non–survivors	***P*** value
	***N*** = 839	***N*** = 489	***N*** = 350	
Age (years)	61 [50 to 70]	58 [47 to 68]	63 [53 to 73]	< 0.001
Gender (male)	555 (66%)	314 (64%)	241 (69%)	0.23
Ethnicity, no. (%)				0.09
African	12 (1%)	5 (1%)	7 (2%)	
Asian	9 (1%)	4 (1%)	5 (1%)	
Caucasian	733 (87%)	428 (88%)	305 (87%)	
Latin American	16 (2%)	8 (2%)	8 (2%)	
Other	69 (8%)	44 (9%)	25 (7%)	
BMI (kg/m^2^)	25 [23 to 29]	25 [23 to 29]	25 [23 to 28]	0.61
PBW (kg)	71 [62 to 75]	71 [62 to 75]	71 [62 to 75]	0.66
ARDS, *N* (%)	223 (22%)	130 (21%)	93 (22%)	0.96
Mild	118 (14%)	67 (14%)	51 (14%)	
Moderate	97 (12%)	60 (13%)	37 (10%)	
Severe	8 (1%)	3 (1%)	5 (1%)	
Reason for ICU admission, no. (%)				0.25
Planned surgery	116 (14%)	75 (15%)	41 (12%)	
Emergency surgery	207 (25%)	122 (25%)	85 (24%)	
Medical	513 (61%)	289 (59%)	224 (64%)	
Risk of death (%)^a^	32 [14 to 59]	23 [10 to 47]	48 [23 to 74]	< 0.001
Reason for intubation, no. (%)				0.06
Cardiac arrest	134 (16%)	58 (12%)	76 (22%)	
Post–surgery	207 (25%)	130 (27%)	77 (22%)	
Depressed level of consciousness	77 (9%)	45 (9%)	32 (9%)	
Respiratory failure	312 (37%)	187 (38%)	125 (36%)	
Other	109 (13%)	69 (14%)	40 (11%)	
Chronic comorbidity, no. (%)				
Hypertension	231 (28%)	137 (28%)	94 (27%)	0.73
Diabetes mellitus	127 (15%)	69 (14%)	58 (17%)	0.40
Heart failure	69 (8%)	44 (9%)	25 (5%)	0.39
Chronic kidney failure	60 (7%)	19 (4%)	41 (12%)	< 0.001
Cirrhosis	9 (1%)	1 (0%)	8 (2%)	0.01
COPD	89 (11%)	43 (9%)	46 (13%)	0.06
Oxygen at home	5 (1%)	2 (0%)	3 (1%)	0.72
Cancer	46 (5%)	19 (4%)	27 (8%)	0.43
Immunodeficiency	88 (10%)	47 (10%)	41 (12%)	0.40
Ventilation at home	10 (1%)	6 (1%)	4 (1%)	1.00
Vital signs				
SpO_2_ (%)	98 [96 to 100]	98 [96 to 100]	98 [96 to 100]	0.79
Heart rate (bpm)	101 [83 to 120]	100 [83 to 118]	102 [83 to 121]	0.39
MAP (mm Hg)	57 [52 to 62]	58 [53 to 63]	56 [50 to 60]	< 0.001
Temperature (C)	36.7 [35.9 to 37.3]	36.8 [36.0 to 37.4]	36.6 [35.7 to 37.2]	0.03
Severity of illness, SOFA score				
Total	5 [4 to 8]	4 [3 to 7]	6 [4 to 9]	< 0.001
Non–pulmonary SOFA	4 [4 to 7]	4 [3 to 6]	5 [4 to 8]	< 0.001
Pulmonary	0 [0 to 1]	0 [0 to 1]	0 [0 to 1]	0.07
Hematologic	0 [0 to 1]	0 [0 to 1]	0 [0 to 2]	0.12
Liver	0 [0 to 0]	0 [0 to 0]	0 [0 to 0]	0.12
Circulation	4 [3 to 4]	3 [2 to 4]	4 [3 to 4]	< 0.001
Neurology	0 [0 to 1]	0 [0 to 1]	0 [0 to 3]	0.01
Renal	0 [0 to 0]	0 [0 to 0]	0 [0 to 1]	< 0.001
Outcomes				
ICU mortality, no. (%)	220 (26%)	0 (0%)	220 (63%)	< 0.001
Hospital mortality, no. (%)	305 (36%)	3 (1%)	301 (86%)	< 0.001
ICU LOS, days	7 [4 to 12]	7 [4 to 13]	7 [4 to 12]	0.12
Hospital LOS, days	22 [11 to 42]	31 [17 to 50]	13 [6 to 27]	< 0.001
Duration of mechanical ventilation, days	5 [3 to 10]	5 [3 to 10]	6 [3 to 10]	0.60
VFD–28, days	17 [0 to 24]	23 [18 to 25]	0 [0 to 0]	< 0.001
Ventilatory variables, median [IQR]				
Tidal volume (ml)	471 [410 to 540]	476 [ 417 to 546]	461 [405 to 531]	0.04
Tidal volume (ml/kg PBW)	6.8 [6.0 to 8.0]	6.9 [6.1 to 7.9]	6.8 [5.8 to 7.9]	0.09
Respiratory rate (bpm)	20 [16 to 26]	20 [15 to 25]	22 [17 to 28]	0.003
FiO_2_ (%)	40 [40 to 48]	40 [40 to 45]	40 [40 to 50]	0.27
P_max_ (cm H_2_O)	23 [18 to 28]	22 [18 to 27]	24 [19 to 30]	< 0.001
PEEP (cm H_2_O)	7 [5 to 10]	7 [5 to 10]	8 [5 to 10]	0.22
Dynamic compliance (ml/cm H_2_O)	31 [23 to 41]	32 [25 to 43]	29 [22 to 37]	< 0.001
ΔP (cm H_2_O)	15 [12 to 19]	15 [12 to 18]	16 [13 to 20]	< 0.001
MP (10^−3^ J/min/kg PBW)	206 [145 to 298]	194 [136 to 280]	218 [156 to 339]	< 0.001
Blood gas analysis results, median [IQR]				
PaO_2_ (mm Hg)	91 [77 to 110]	91 [77 to 109]	92 [78 to 112]	0.68
PaCO_2_ (mm Hg)	37 [41 to 43]	38 [32 to 43]	37 [31 to 43]	0.53
pHa	7.38 [7.33 to 7.44]	7.39 [7.35 to 7.44]	7.38 [7.32 to 7.43]	< 0.001
Bicarbonate (mmol/l)	23 [20 to 27]	24 [21 to 27]	22 [19 to 26]	< 0.001

Abbreviations: *ΔP* respiratory system driving pressure, *ARDS* acute respiratory distress syndrome, *BMI* body mass index, *COPD* chronic obstructive pulmonary disease, *FiO*_*2*_ fraction of inspired oxygen, *ICU* intensive care unit, *MAP* mean arterial pressure, *MP* mechanical power of ventilation normalized for predicted bodyweight, *PaCO*_*2*_ arterial carbon dioxide tension, *PaO*_*2*_ arterial oxygen tension, *PBW* predicted body weight, *PEEP* positive end-expiratory pressure, *P*_*max*_ maximum airway pressure; *SOFA* sepsis-related organ failure assessment score, *SpO*_*2*_ peripheral pulse oximetry, *LOS* length of stay, *VFD–28* days ventilator-free and alive

^a^Risk of death is based on the APACHE IV score

### ORs for major outcomes

The OR for 90-day mortality for an increase of 1 cm H_2_O in the modified ΔP was 1.05 [95% confidence interval (CI) 1.03 to 1.08]; *P* < 0.001; the OR for 90-day mortality for an increase of 100 10^−3^ J/min/kg PBW in the MP was 1.20 [95% CI 1.09 to 1.33]; *P* < 0.001.

For ICU mortality, ORs were (modified ΔP) 1.10 [95% CI 1.07 to 1.13]; *P* < 0.001 and (MP) 1.35 [95% CI 1.21 to 1.51]; *P* < 0.001. For hospital mortality, ORs were (modified ΔP) 1.07 [95% CI 1.04 to 1.10]; *P* < 0.001 and (MP) 1.26 [95% CI 1.14 to 1.41]; *P* < 0.001. The EE for VFDs for 1 cm H_2_O increase were (modified ΔP) −0.4 [95% CI −0.6 to −0.3]; *P* < 0.001 and (MP) −1.6 [95% CI −2.2 to −1.0]; *P* < 0.001.

### Predictive validity

**Fig. 3:**
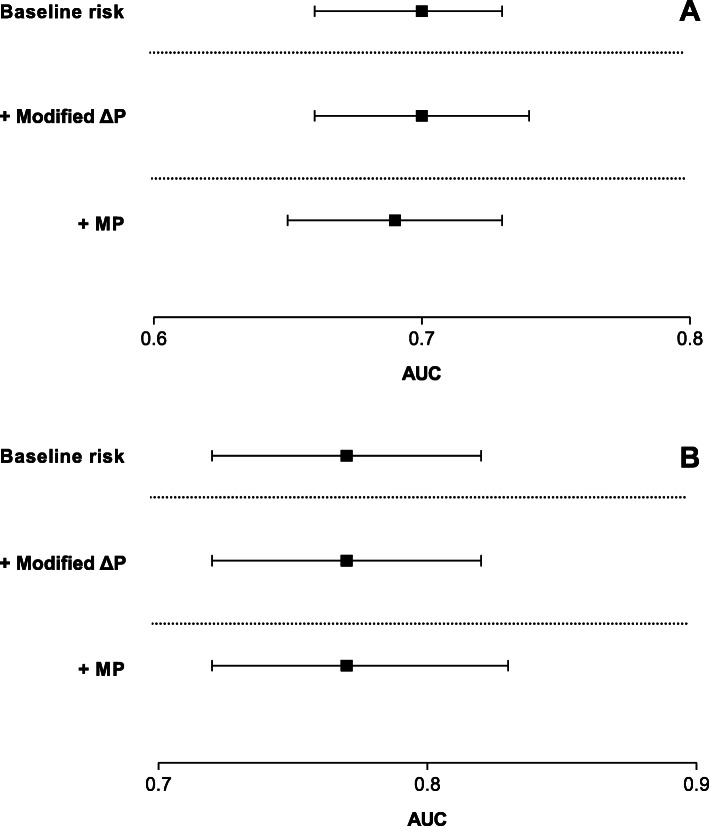
(**a**) Predictive validity for the ΔP and the MP compared to baseline risk based on APACHE IV scores; (**b**) predictive validity for the ΔP and the MP compared to baseline risk based on SAPS II. Abbreviations: ΔP, respiratory system driving pressure; ARDS, acute respiratory distress syndrome; ICU, intensive care unit; MP, mechanical power of ventilation normalized for predicted body weight; PBW, predicted body weight

Discrimination did not improve risk of death classification when adding the modified ΔP or the MP to the APACHE IV score. Discrimination did also not improve risk of death classification when adding the modified ΔP or the MP to the SAPS II (Fig. 3). Furthermore, discrimination for death did not improve when adding the combination of modified ΔP and MP to APACHE IV score or the SAPS II.

### Sensitivity analyses

There was no significant interaction between the predictive validity of the modified ΔP (*P =* 0.10) and the MP (*P =* 0.83) for 90-day mortality, and the presence of ARDS, meaning that the presence of ARDS did not affect the poor predictive validity of the modified ΔP and the MP found for the whole cohort.

## Discussion

The results of this post hoc analysis of a large cohort of ICU patients under controlled invasive ventilation for > 24 h for acute respiratory failure can be summarized as follows: (1) classification of patients into groups based on high or low modified ΔP and MP at 24 h after start of invasive ventilation results in groups with a difference in risk for 90-day mortality, (2) discrimination using the APACHE IV score or SAPS II for 90-day mortality does not improve by adding the modified ΔP or the MP, and (3) there is no significant interaction between the predictive validity of the modified ΔP or the MP and the presence of ARDS. While predicting outcomes of patients who are under invasive ventilation for > 24 h has the potential to facilitate identification of patients that may benefit from specific management strategies or closer monitoring as well as optimizing the selection of inclusion criteria in future studies, the current findings argue against using the modified ΔP or the MP for that purpose.

The strengths of this study are the use of prospectively collected ventilation and outcome data that were captured by a team of dedicated researchers who were trained to use only ventilator settings and ventilator parameters in stable situations. Furthermore, as patients who had or may have had spontaneous breathing activity were excluded, reliable calculations of the modified ΔP and the MP could be used. The data and outcome assessors were also trained in using strict diagnostic criteria and confirmed outcome data. Also, in MARS patients were enrolled over a relatively short period, minimizing the influence of changes in clinical practice. MARS itself had no exclusion criteria, increasing the generalizability of the findings of the current study. Finally, the number of patients who were excluded because of missing data was minimal.

Parameters focusing on lung mechanics, such as the ΔP and the MP, are receiving increasing interest. The modified ΔP is associated with outcome and is even suggested to be the only ventilatory parameter that is independently associated with outcome [9‑13]. The finding of the current analysis that classification using a high or low modified ΔP results in groups with a clear difference in mortality rates; however, this did not translate into improved discrimination for clinically relevant outcomes. The results of classification using the MP are also in line with the results of a recent meta-analysis in patients with ARDS, were having a high MP was associated with higher mortality [16]. However, also the MP had no predictive validity for the clinical outcomes used in this analysis.

Several limitations should be acknowledged. This was a post hoc analysis, performed in a selected subgroup of patients because of the strict inclusion of patients who still received controlled invasive ventilation at 24 h of ventilation. Because patients in here analyzed cohort were exclusively receiving pressure-controlled ventilation, the calculation of the modified ΔP had to be adapted, i.e., *P*_max_ was used instead of *P*_peak_, as with pressure-controlled ventilation there is no *P*_peak_. Of note, *P*_max_ was measured and reported at zero flow in patients without a spontaneous breathing effort [22, 24]. Furthermore, and for the same reasons, also the “power equation” was adapted. Also, excluding patients in whom RR_set_ was lower than RR_measured_ could have led to exclusion of patients with auto triggering. Finally, the use of set PEEP instead of measured PEEP for the calculation of the ΔP could have led to an overestimation of the ΔP. Therefore, we refer to the modified ΔP instead of ΔP.

## Conclusion

In this conveniently sized cohort of ICU patients under invasive ventilation for > 24 h, 90-day mortality as well as other clinical outcomes differed between patients with low or high modified ΔP and MP. The modified ΔP and the MP at 24 h after the start of invasive ventilation, however, had poor predictive validity, there was no interaction for patients with and without ARDS.

## Data Availability

The datasets used and/or analyzed during the current study are available from the corresponding author on reasonable request.

## Abbreviations

ΔP: Driving pressure
APACHE: Acute Physiology, Age and Chronic Health Evaluation
ARDS: Acute respiratory distress syndrome
AUROC: Area under the receiver operator characteristic curve
BMI: Body mass index
C: Respiratory system compliance
COPD: Chronic obstructive pulmonary disease
CI: Confidence interval
EE: Effect estimate
Eq: Equation
FiO_2_: Fraction of inspired oxygen
ICU: Intensive care unit
LOS: Length of stay
MARS: Molecular diagnosis and risk stratification of sepsis
MP: Mechanical power normalized for predicted body weight
OR: Odds ratio
PaCO_2_: Arterial carbon dioxide tension
PaO_2_: Arterial oxygen tension
PBW: Predicted body weight
PEEP: Positive end-expiratory pressure
*P*_max_: Maximum airway pressure
*P*_peak_: Peak pressure
*P*_plat_: Plateau pressure
RR: Respiratory rate
RR_measured_: Measured respiratory rate
RR_set_: Set respiratory rate
SAPS: Simplified Acute Physiology Score
SpO_2_: Peripheral pulse oximetry
SOFA: Sepsis-related organ failure assessment score
VFD–28: Days ventilator-free and alive
*V*_T_: Tidal volume
